# Chronic Kidney Disease and Its Impact on a Prothrombotic State in Patients with Atrial Fibrillation

**DOI:** 10.3390/jcm9082476

**Published:** 2020-08-01

**Authors:** Paweł T. Matusik, Zbigniew Heleniak, Elżbieta Papuga-Szela, Krzysztof Plens, Jacek Lelakowski, Anetta Undas

**Affiliations:** 1 Jagiellonian University Medical College, Faculty of Medicine, Institute of Cardiology, 80 Prądnicka Street, 31-202 Kraków, Poland; pawel.matusik@uj.edu.pl (P.T.M.); jacek.lelakowski@uj.edu.pl (J.L.); 2 The John Paul II Hospital, 80 Prądnicka Street, 31-202 Kraków, Poland; 3 Medical University of Gdańsk, Department of Nephrology, Transplantology and Internal Medicine, 7 Dębinki Street, 80-952 Gdańsk, Poland; zbigniew.heleniak@gumed.edu.pl; 4 The John Paul II Hospital, Immunology Clinic, 80 Prądnicka Street, 31-202 Kraków, Poland; cardin@tlen.pl; 5 KCRI, 5B Miechowska Street, 30-055 Kraków, Poland; plens_krzysztof@o2.pl

**Keywords:** atrial fibrillation, chronic kidney disease, prothrombotic state, thrombin potential, clot properties, fibrinolysis, inflammation

## Abstract

It is unclear whether chronic kidney disease (CKD) increases thromboembolism in atrial fibrillation (AF). We conducted a retrospective cross-sectional analysis of 502 non-anticoagulated AF patients (median age, 66 (60–73) years, median CHA_2_DS_2_-VASc score, 3.0 (2.0–4.0)) with an estimated glomerular filtration rate (eGFR) ≥ 15 mL/min/1.73 m^2^. Endogenous thrombin potential (ETP), clot permeability (K_s_), and clot lysis time (CLT), among others, were determined. Patients with stage 4 CKD (n = 87; 17.3%) had higher ETP and prolonged CLT compared with those with stage 3 CKD. In patients with stages 3 to 4 CKD (n = 180; 35.9%) N-terminal pro-B-type natriuretic peptide predicted low K_s_ (the lowest quartile, odds ratio [OR] per 100 pg/mL: 1.03, 95% confidence interval [CI]: 1.01–1.06) and prolonged CLT (the top quartile, OR per 100 pg/mL: 1.05, 95% CI: 1.02–1.08), but not high ETP. In the whole cohort, after adjustment for CHA_2_DS_2_-VASc score, stage 4 CKD, but not stage 3 CKD, predicted high ETP (OR: 9.06; 95% CI: 4.44−18.46) and prolonged CLT (OR: 3.58; 95% CI: 1.76–7.28), but not low K_s_. compared to the reference eGFR category. This study is the first to demonstrate the prothrombotic and antifibrinolytic alterations in AF patients with stage 4 CKD, but not stage 3 CKD irrespective of clinical stroke risk factors.

## 1. Introduction

Chronic kidney disease (CKD) is associated with poor prognosis [1]. At the same time in patients with atrial fibrillation (AF) impaired renal function is independently related to stroke or systemic embolism [2]. The modified scoring systems in AF patients incorporating extra points for renal impairment to the congestive heart failure, hypertension, age (≥75 years), diabetes, stroke or transient ischemic attack (CHADS_2_) or the congestive heart failure, hypertension, age (≥75 years), diabetes, stroke or transient ischemic attack or systemic embolism, vascular disease, age (65–74 years), sex (female) (CHA_2_DS_2_-VASc) scores to enhance their clinical performance have been proposed, however, several studies assessing a clinical value of the modified scores yielded inconsistent findings [2,3,4,5,6].

It is well established that a prothrombotic state occurs in AF [7,8]. Patients with AF have been reported to show increased fibrin clot density and decreased clot lysability [8]. These unfavorable fibrin clot properties, the so-called prothrombotic clot phenotype, have been found to be associated with N-terminal pro-B-type natriuretic peptide (NT-proBNP) and growth differentiation factor-15 [9,10]. Recently, it has been demonstrated that low plasma clot permeability and prolonged clot lysis can predict ischemic stroke or transient ischemic attack in AF during follow-up [11,12].

Inconsistent data have suggested that CKD, in particular stage 5, also leads to a prothrombotic state [13,14]. In patients with severe or end-stage CKD increased thrombin generation has been shown by Sagripanti et al. [15], in contrast to another study which reported no difference in endogenous thrombin potential (ETP) between stage 5 CKD and healthy controls [13]. Increased fibrin clot density has been reported in stage 4 CKD compared with stage 1 CKD [16], like in haemodialysis patients in whom impaired fibrinolysis has also been demonstrated [14]. Higher levels of von Willebrand factor and increased fibrin formation have been found in haemodialysis patients compared to controls [17]. Thrombotic and hemostatic alterations in patients with CKD at least in part result from the uremic milieu associated with accumulation of uremic toxins [18]. However, most AF studies focused on prothrombotic abnormalities excluded patients with stage 4 CKD or their number was small [9,10,11,12,16].

In the current study, we sought to test a hypothesis that in AF patients with stage 3 or 4 CKD increased thrombin generation and unfavorable fibrin clot properties can be observed independently of clinical stroke risk factors.

## 2. Materials and Methods

### 2.1. Patients

We performed a retrospective cross-sectional analysis involving 502 patients with AF. Included patients had estimated glomerular filtration rate (eGFR) ≥15 mL/min/1.73 m^2^ according to the Chronic Kidney Disease Epidemiology Collaboration (CKD-EPI) equation and were recruited from inpatient and outpatient settings [19]. The subgroup of this cohort was reported in our previous studies [9,20]. The exclusion criteria were: known cancer, liver cirrhosis, signs or symptoms of acute infection, recent cardiovascular event, current anticoagulation, coagulation abnormalities, i.e., elevated activated partial thromboplastin time >40 s or international normalized ratio >1.3 (resulting from vitamin K antagonist use).

We collected demographic and clinical data. Types of AF and risk factors were defined according to the European Society of Cardiology guidelines [10]. Stage 3 and 4 CKD were defined as eGFR 30-59 mL/min/1.73 m^2^ and 15–29 mL/min/1.73 m^2^, respectively. We calculated the CHA_2_DS_2_-VASc score [21], the R-CHA_2_DS_2_-VASc and R_2_-CHA_2_DS_2_-VASc scores by addition of one or two points, respectively, for the presence of eGFR < 60 mL/min/1.73 m^2^ [4,22]. Elevated C-reactive protein (CRP) was defined as >3 mg/L.

Studies included into current analysis were approved by the local ethics committee and all study participants gave informed consent. Research conformed to the ethical guidelines of the Declaration of Helsinki. The STROBE Statement was used to guide reporting in our study.

### 2.2. Laboratory Tests

Fasting blood samples were taken off anticoagulation with minimal stasis from the antecubital vein. In patients on oral anticoagulants, who were switched to a low-molecular-weight heparin, blood was drawn minimum 12 h since the last heparin injection. Patients receiving dabigatran, rivaroxaban, or apixaban were drawn after at least 24 h since the last dose. Routine laboratory investigations were assessed by standard laboratory techniques. Fibrinogen was determined using the Clauss method. NT-proBNP was assessed using electrochemiluminescence immunoassay (Roche Diagnostics, Mannheim, Germany). Plasminogen activator inhibitor-1 (PAI-1) antigen was measured by the enzyme-linked immunosorbent assay (American Diagnostica, Stamford, CT, USA).

ETP was assessed using a fluorometer (Ascent Reader, Thermolat Systems OY, Helsinki, Finland) by calibrated automated thrombography (Thrombinoscope BV, Maastricht, the Netherlands), as previously reported [9,10,23,24,25].

Clot properties were determined in citrated plasma (volume-to-volume ratio, ratio of 9:1 of 3.2% sodium citrate) in duplicate, as previously described [9,10,26]. Fibrin clot permeability was measured as described previously [27]. The permeation coefficient (K_s_), reflecting the average size of fiber network pores (a measure of fibrin network density), was calculated using the following equation:

K_s_ = Q × L × ƞ / t × A × Δp, (1)

where Q is the flow rate in time, A indicates the cross-sectional area in cm^2^, L is the length of a fibrin gel, ƞ is the viscosity of liquid in poise, t is the percolating time, while Δp indicates a differential pressure in dyne/cm^2^.

Clot lysis time (CLT), reflecting fibrin clot susceptibility to lysis, was determined as described elsewhere [27]. CLT following addition to plasma of 0.6 pM human tissue factor (Innovin, Siemens, Marburg, Germany), 12 µmol/L phospholipid vesicles and 60 ng/mL recombinant tissue-type plasminogen activator (Boehringer Ingelheim, Ingelheim, Germany) was determined from the midpoint of the clear-to-maximum-turbid transition to the midpoint of the maximum-turbid-to-clear transition [9,10]. All measurements were performed by technicians blinded to the origin of the samples. Intra-assay and inter-assay coefficients of variation were 5–7%.

### 2.3. Statistical Analysis

Assuming a difference between eGFR categories of at least 15% the study would require a sample size of 74 for each group (assuming equal group sizes), to have a 80% chance of detecting a difference at a two-sided *p-*value of 5%, based on the reported values of ETP [13].

Continuous variables were expressed as the means ± standard deviations or medians with interquartile ranges. Normality of the data was assessed by the Shapiro–Wilk test, while equality of variances by the Levene’s test. Student’s or Welch’s t-test (for normally distributed variables, depending on the equality of variances) or Mann–Whitney U test (for non-normally distributed variables), were used to compare continuous variables between two groups. Differences between more than two groups were compared using the classical one-way ANOVA or the Welch’s ANOVA (depending on the equality of variances), while the Kruskal–Wallis test was used for non-normally distributed continuous variables. Pairwise comparisons were performed with Tukey’s HSD test or with Kruskal–Wallis test with *p*-values adjusted by the Bonferroni correction. Associations between continuous variables were tested using a Pearson correlation coefficient or Spearman’s rank correlation coefficient, as appropriate. Categorical variables were shown as numbers and percentages and were compared by the Pearson’s chi-squared test or the Fisher’s exact test, as appropriate, while the comparison of nominal variables among four eGFR categories was performed using Cochran–Armitage test for trend. Logistic regression analysis was performed to assess whether eGFR categories are independent predictors of high ETP (the top quartile), low K_s_ (the lowest quartile), and prolonged CLT (the top quartile). Potential confounders were fibrinogen, CHA_2_DS_2_-VASc score and risk factors of the CHA_2_DS_2_-VASc score (including age as a categorical variable: <65 vs. 65-74 vs. ≥75 years). The results are presented as odds ratios (OR) with 95% confidence intervals (CI).

Two-sided *p-*values < 0.05 were considered statistically significant. Statistical analyses were performed with JMP^®^, version 14.2.0 (SAS Institute Inc., Cary, NC, USA) and IBM SPSS Statistics for Windows, version 25 (IBM Corp., Armonk, NY, USA).

## 3. Results

### 3.1. Patients

The study group involved 502 AF patients (men, 51.2%, median age 66.0 years) with a median CHA_2_DS_2_-VASc score of 3.0 (Table 1). Patients with stages 3 to 4 CKD (n = 180, 35.9%) were older and had more commonly diabetes and vascular disease (51.1% vs. 29.8%, *p* < 0.0001), but prior cerebrovascular events or systemic embolism were less frequent, compared with the remaining subjects (18.0% vs. 9.4%, *p* = 0.01). The former group had higher CHA_2_DS_2_-VASc, R-CHA_2_DS_2_-VASc, and R_2_-CHA_2_DS_2_-VASc scores, with no differences in any of the 3 scores between stage 3 and stage 4 CKD (Table 1). In the whole group, eGFR inversely correlated with CHA_2_DS_2_-VASc (R = −0.28), R-CHA_2_DS_2_-VASc (R = −0.46), and R_2_-CHA_2_DS_2_-VASc scores (R = −0.59, all *p* < 0.0001).

### 3.2. Markers of a Prothrombotic State

**Figure 1:**
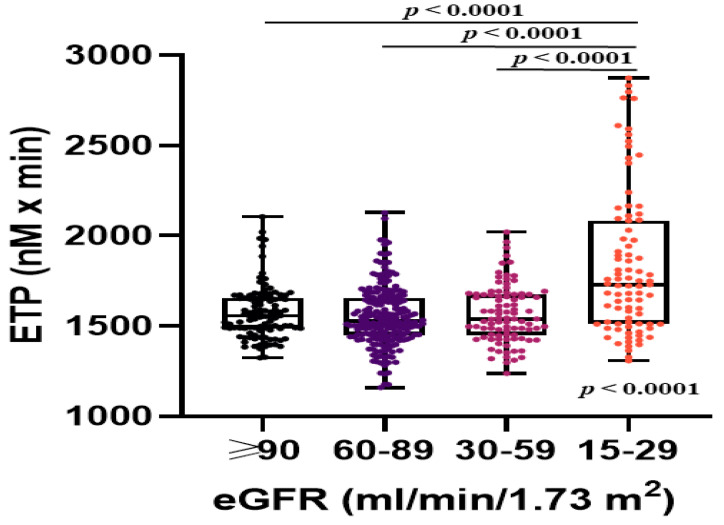
Associations between endogenous thrombin potential (ETP) and estimated glomerular filtration rate (eGFR) categories in patients with atrial fibrillation. Data are shown as medians (interquartile ranges) and maximum and minimum values.

Patients with stages 3 to 4 CKD had higher ETP compared with the remainder (Table 2). Patients with stage 4 CKD had increased ETP when compared to all other eGFR categories (all *p* < 0.0001; Figure 1). ETP weakly correlated with eGFR (R = −0.18, *p* < 0.0001). The impact of CKD on high ETP was largely driven by stage 4 CKD (Table 3). Analysis of various clinical stroke risk factors revealed that in patients with eGFR < 60 mL/min/1.73 m^2^ heart failure/LV dysfunction was associated with lower ETP, while other showed no association. In patients with stages 3 to 4 CKD high ETP (the top quartile, ≥1782.0 nM × min) was only predicted by stage 4 CKD (OR: 7.86, 95% CI: 3.39–18.22, *p* < 0.0001) and heart failure/LV dysfunction (OR: 0.39, 95% CI: 0.17–0.87, *p* = 0.02), while other CHA_2_DS_2_-VASc score risk factors and NT-proBNP were not associated with high ETP.

**Figure 2:**
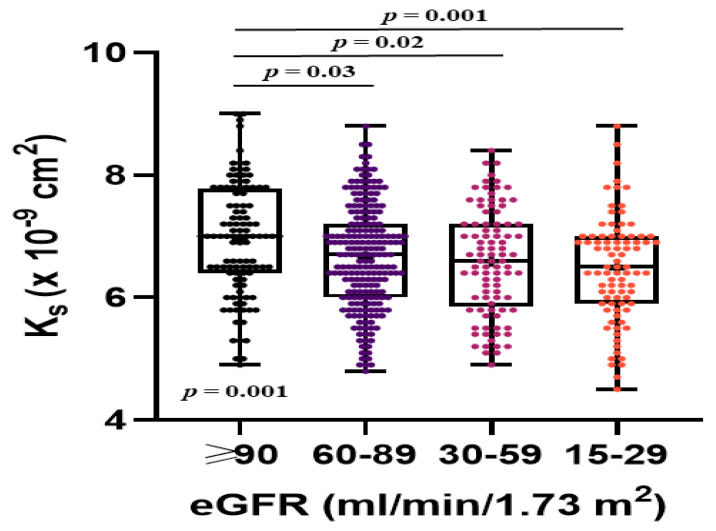
Associations between fibrin clot permeability (K_s_) and estimated glomerular filtration rate (eGFR) categories in patients with atrial fibrillation. Data are shown as medians (interquartile ranges), maximum and minimum values. Mean values are presented as the plus signs.

Patients with stages 3 to 4 CKD had lower K_s_ compared with those with higher eGFR (Table 2, Figure 2), with no difference between stage 3 and stage 4 CKD. K_s_ showed weak inverse associations with CHA_2_DS_2_-VASc, R-CHA_2_DS_2_-VASc, and R_2_-CHA_2_DS_2_-VASc scores (R = −0.17, R = −0.19, and R = −0.20, respectively, all *p* ≤ 0.0001). Any of the decreased eGFR categories did not predict low K_s_ (Table 4). In AF patients with stages 3 to 4 CKD none of the CHA_2_DS_2_-VASc score risk factors was associated with K_s_. In patients with stages 3 to 4 CKD low K_s_ (the lowest quartile, ≤5.90 × 10^−9^ cm^2^) was predicted by NT-proBNP (OR per 100 pg/mL: 1.03, 95% CI: 1.01–1.06, *p* = 0.009).

**Figure 3:**
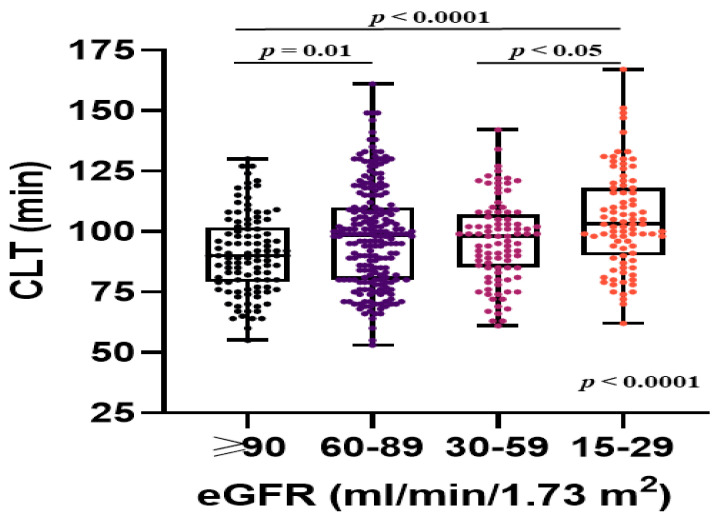
Associations between clot lysis time (CLT) and estimated glomerular filtration rate (eGFR) categories in patients with atrial fibrillation. Data are shown as medians (interquartile ranges) and maximum and minimum values.

In patients with stages 3 to 4 CKD compared with the remainder, we observed impaired fibrinolysis capacity, as evidenced by longer CLT, and there was a slight prolongation of CLT in stage 4 compared with stage 3 CKD (Table 2, Figure 3). The eGFR 15–29 mL/min/1.73 m^2^ predicted prolonged CLT also after adjustment for CHA_2_DS_2_-VASc score risk factors (Table 5). Among the CHA_2_DS_2_-VASc score risk factors, only prior thromboembolic events in AF patients with stages 3 to 4 CKD were associated with CLT. In patients with eGFR <60 mL/min/1.73 m^2^ prolonged CLT (the top quartile, ≥110.8 min) was predicted by stage 4 CKD (OR: 2.41, 95% CI: 1.20–4.84, *p* = 0.01) and NT-proBNP (OR per 100 pg/mL: 1.05, 95% CI: 1.02–1.08, *p* = 0.0002).

There was a tendency towards higher PAI-1 in patients with stages 3 to 4 CKD, compared with the remainder (Table 2). Patients with stage 4 CKD had 34.0% higher PAI-1 than those with stage 3 CKD (*p* = 0.008). PAI-1 correlated weakly with ETP (R = 0.25, *p* < 0.0001) and CLT (R = 0.19, *p* < 0.0001).

### 3.3. Association with Inflammation

Patients with stages 3 to 4 CKD had higher CRP compared with those with eGFR ≥60 mL/min/1.73 m^2^ (Table 2). CRP weakly correlated with ETP (R = 0.15, *p* = 0.006), K_s_ (R = −0.13, *p* = 0.02), and CLT (R = 0.13, *p* = 0.02). Patients with elevated CRP levels (26.2%) had higher ETP (1569.0 [1486.3–1679.5] vs. 1511.0 [1428.0–1619.5] nM × min, *p* = 0.002), decreased K_s_ (6.6 [5.9–7.2] vs. 6.9 [6.3–7.4] × 10^−9^ cm^2^, *p* = 0.02), and prolonged CLT (99.0 [89.0–110.3] vs. 95.0 [79.8–108.0] min, *p* = 0.01). CRP did not correlate with eGFR or any of the 3 risk scores.

## 4. Discussion

To our knowledge, this study is the first to comprehensively assess thrombin generation potential along with plasma fibrin clot density and lysability in AF patients with stages 3 to 4 CKD. Our findings demonstrate that non-anticoagulated AF patients with stage 4 CKD, but not stage 3 CKD, display enhanced prothrombotic state, including increased thrombin formation and impaired fibrinolysis as compared to those with higher eGFR. This suggests that stage 4 CKD is a prothrombotic and antifibrinolytic factor among AF patients. We found that increased ETP represents the most specific feature of the prothrombotic state in AF patients with impaired renal function, also after adjustment for fibrinogen and CHA_2_DS_2_-VASc score risk factors. The present study provides evidence that despite associations between eGFR, some comorbidities and prothrombotic markers in AF patients, stage 4 CKD, but not stage 3, enhances the prothrombotic state in AF.

ETP reflects the capacity of plasma to generate thrombin after in vitro activation of coagulation. Thrombin formation depends on the levels of tissue factor, prothrombin, antithrombin, and the function of protein C pathway [23,24]. Enhanced thrombin formation has been shown to be related to stroke risk factors and AF [7,15,23]. However, lower ETP, which was associated with decreased procoagulant microparticles, has been previously reported in heart failure with reduced ejection fraction [28]. Increased ETP in our patients with AF and stage 4 CKD is in line with observations of higher levels of prothrombin fragment 1 + 2, a marker of thrombin formation in vivo, in patients with severe or end-stage chronic renal failure [15]. However, there have been small studies in which there were no differences in thrombin generation between healthy controls and patients with stages 3–5 CKD, who were free of AF and hemodialysis therapy [13,29]. These differences might suggest that AF and its risk factors increase thrombin generation capacity in severely impaired renal function in part due to endothelial cell damage and increased tissue factor expression leading to heightened prothrombin activation supported by lower antithrombin activity [2,15,23,30]. Importantly, indolic uremic solutes, which accumulate notably in patients with stages 4 to 5 CKD, have been found to elevate tissue factor expression and decrease its degradation [18]. Taken together, the present study supports the view that the capacity of thrombin formation is markedly increased in patients in whom AF coexists with stage 4 CKD, highlighting the need for appropriate anticoagulant therapy in this subgroup.

This study is the first to show that in non-anticoagulated AF patients impaired renal function (eGFR < 60 mL/min/1.73 m^2^) is potent enough to affect complex processes governing formation of fibrin clot network and the key measure of its density, permeability [31]. Low fibrin clot permeability reported here in non-anticoagulated AF patients with low eGFR is consistent with the results of Lau et al. [16] who demonstrated increased fibrin clot density in anticoagulated AF patients from stage 1 CKD to stage 4 CKD and those of Sjøland et al. [32] in patients with end-stage renal disease on peritoneal dialysis. Denser clot formation is likely related to post-translational fibrinogen modifications in particular its carbonylation at the state of enhanced oxidative stress in CKD and AF, due to both antioxidant depletions and increased reactive oxygen species production [8,31,33,34]. Elevated markers of oxidative stress have been reported to be associated with decreased fibrin clot permeability in end-stage renal disease [14]. The impact of fibrin oxidative modifications on thrombotic tendency in AF patients with CKD remains to be explored.

We provided new insights into altered fibrinolysis observed in AF [8], by showing that AF patients with stage 4 CKD are characterized by impaired fibrinolysis compared to those with higher eGFR. This suggests that compensatory mechanisms are inefficient to control efficiency of lysis in subjects with AF and eGFR below 30 mL/min/1.73 m^2^, while stage 3 CKD appears not to affect fibrinolysis in AF compared to patients with eGFR 60–89 mL/min/1.73 m^2^ or higher. Since PAI-1, a major inhibitor of fibrinolysis, is the key determinant of CLT [7], our study confirms that this effect also occurs in AF patients with CKD. Furthermore, impaired fibrinolysis in patients with CKD and AF could be in part attributable to oxidative fibrinogen/fibrin modifications, which is supported by the finding for hemodialysis patients indicating that increased oxidative stress is associated with prolonged clot lysis [14]. Multiple mechanisms underlying hypolysability in severe CKD require further investigation.

A novel observation is that in AF patients with stages 3 to 4 CKD NT-proBNP can predict a prothrombotic state, which is in line with our previous studies in AF patients [9,10]. In the current study we observed also that in AF elevated CRP was associated with higher ETP, which supports growing evidence on association of inflammation with thrombogenesis [7,10,35]. Elevation of NT-proBNP and cardiac troponin in CKD, which results at least in part from decreased renal elimination indicates another possible shared cardiac and kidney pathophysiological pathway that is implicated in increased risk of stroke both in AF and CKD [36]. Thus, our data might be considered as an additional argument for benefits from the determination of biomarkers for better stroke risk assessment in AF patients [35].

Prevention of thromboembolic events in patients with AF and severe CKD is a complex issue. Appropriate anticoagulant therapy and frequent renal function monitoring are crucial in these patients [37]. Moreover, taking into account concomitant increased risk of bleeding in patients with CKD and reported kidney injury in patients on warfarin during excessive anticoagulation non-vitamin K oral anticoagulants (unless contraindicated), especially apixaban, could be preferred over vitamin K antagonists [38,39,40,41,42]. Left atrial appendage occlusion might be an option in patients with severe CKD who have contraindications to lifelong anticoagulant therapy [43]. It might be speculated that a prothrombotic state in patients with AF could be attenuated following sinus rhythm restoration with the use of catheter ablation or cardioversion of AF, however, data on this is questionable, taking into account increased thrombin generation, lower K_s_ and prolonged CLT in patients with paroxysmal and persistent AF in sinus rhythm compared to controls [44]. Moreover, patients with AF, assessed 30 days after cardioversion, compared to healthy controls were characterized by increased levels of fibrin D-dimer, soluble thrombomodulin, and von Willebrand factor, which were similar (expect for lower fibrin D-dimer) to levels observed in patients with permanent AF [45]. The coexistence of severe CKD is likely to enhance prothrombotic alterations after sinus rhythm restoration. Further clinical studies are needed to identify best therapeutic strategy to reduce a prothrombotic state in AF patients with stage 4 CKD.

Several study limitations should be acknowledged. The study was moderate in size but was sufficiently powered to demonstrate the difference in ETP between eGFR categories. Clinical data, fibrin clot properties and ETP were assessed once thus their changes over time cannot be excluded. We did not assess other factors which might affect a prothrombotic state in AF and CKD, e.g., platelet activation and indolic uremic toxins [7]. Moreover, the present results may not be applicable for AF patients with end-stage CKD or patients on haemodialysis, who were not eligible [14,32]. We used the CKD-EPI equation for estimation of eGFR, which was also tested in the context of CHA_2_DS_2_-VASc score in patients with AF [4]. The use of other formulas might slightly and most likely insignificantly changed the size of eGFR categories. Lastly, we did not assess follow-up data. It remains to be established whether prothrombotic state markers in stage 4 CKD may predict clinical outcomes in AF patients.

## 5. Conclusions

In AF patients stage 4 CKD is independently associated with increased thrombin generation and decreased fibrinolysis capacity, irrespective of clinical stroke risk factors. We did not observe similar alterations in AF patients with stage 3 CKD. Our findings suggest that stage 4 CKD should be considered as a potential stroke risk factor in AF, however large cohort studies are needed to validate this hypothesis.

## Figures and Tables

**Table 1:** Patient characteristics stratified by estimated glomerular filtration rate (eGFR) categories.

Variables	Total, n = 502	eGFR Categories		*p* Value	eGFR Categories				*p* Value
		≥ 60 mL/min/1.73 m^2^, n = 322	15–59 mL/min/1.73 m^2^, n = 180		≥ 90 mL/min/1.73 m^2^, n = 108	60–89 mL/min/1.73 m^2^, n = 214	30–59 mL/min/1.73 m^2^, n = 93	15–29 mL/min/1.73 m^2^, n = 87	
**Demographics**									
**Age (years)**	66.0 (60.0–73.0)	63.0 (58.0–70.0)	71.0 (64.0–76.0)	<0.0001	60.0 (55.3–64.0)	65.0 (59.0–72.0)	71.0 (64.0–74.0)	71.0 (65.0–77.0)	<0.0001
**Male sex, n (%)**	257 (51.2)	166 (51.6)	91 (50.6)	0.83	83 (76.9)	83 (38.8)	32 (34.4)	59 (67.8)	0.20
**BMI (kg/m^2^)**	27.9 (25.0– 31.0)	28.0 (24.7–31.4)	27.8 (25.4–30.6)	0.97	28.1 (25.2–31.1)	27.7 (24.6–31.5)	28.7 (25.6–32.5)	27.0 (24.9–29.3)	0.04
**Type of AF and Risk Scores**									
**Permanent AF, n (%)**	140 (27.9)	70 (21.7)	70 (38.9)	<0.0001	38 (35.2)	32 (15.0)	23 (24.7)	47 (54.0)	<0.0001
**Paroxysmal AF, n (%)**	198 (39.4)	156 (48.4)	42 (23.3)		40 (37.0)	116 (54.2)	42 (45.2)	0 (0.0)	
**Persistent AF, n (%)**	164 (32.7)	96 (29.8)	68 (37.8)		30 (27.8)	66 (30.8)	28 (30.1)	40 (46.0)	
**CHA_2_DS_2_-VASc score**	3.0 (2.0–4.0)	2.0 (1.0–4.0)	3.0 (2.0–5.0)	<0.0001	2.0 (1.0–3.0)	2.0 (2.0–4.0)	3.0 (2.0–5.0)	3.0 (2.0–4.0)	<0.0001
**R-CHA_2_DS_2_-VASc score**	3.0 (2.0–5.0)	2.0 (1.0–4.0)	4.0 (3.0–6.0)	<0.0001	2.0 (1.0–3.0)	2.0 (2.0–4.0)	4.0 (3.0–6.0)	4.0 (3.0–5.0)	<0.0001
**R_2_-CHA_2_DS_2_-VASc score**	4.0 (2.0–5.0)	2.0 (1.0–4.0)	5.0 (4.0–7.0)	<0.0001	2.0 (1.0–3.0)	2.0 (2.0–4.0)	5.0 (4.0–7.0)	5.0 (4.0–6.0)	<0.0001
**Comorbidities and CVD Risk Factors**									
**Hypertension, n (%)**	265 (52.8)	165 (51.2)	100 (55.6)	0.35	52 (48.1)	113 (52.8)	49 (52.7)	51 (58.6)	0.18
**Heart failure / LV dysfunction, n (%)**	156 (31.1)	94 (29.2)	62 (34.4)	0.22	21 (19.4)	73 (34.1)	42 (45.2)	20 (23.0)	0.26
**Diabetes mellitus, n (%)**	110 (21.9)	59 (18.3)	51 (28.3)	0.009	17 (15.7)	42 (19.6)	21 (22.6)	30 (34.5)	0.002
**Previous MI, n (%)**	101 (20.1)	59 (18.3)	42 (23.3)	0.18	20 (18.5)	39 (18.2)	23 (24.7)	19 (21.8)	0.31
**History of smoking, n (%)**	152 (30.3)	111 (34.5)	41 (22.8)	0.006	52 (48.1)	59 (27.6)	26 (28.0)	15 (17.2)	<0.0001

Data are presented as median (interquartile range) or number (percentage). AF, atrial fibrillation; BMI, body mass index; CVD, cardiovascular disease; LV, left ventricular; MI, myocardial infarction; n, number.

**Table 2:** Laboratory parameters stratified by estimated glomerular filtration rate (eGFR) categories.

Variables	Total, n = 502 *	eGFR Categories		*p* Value	eGFR Categories				*p* Value
		≥ 60 mL/min/1.73 m^2^, n = 322	15–59 mL/min/1.73 m^2^, n = 180		≥ 90 mL/min/1.73 m^2^, n = 108	60–89 mL/min/1.73 m^2^, n = 214	30–59 mL/min/1.73 m^2^, n = 93	15–29 mL/min/1.73 m^2^, n = 87	
**Laboratory Tests Results**									
**eGFR** **(ml/min/1.73 m^2^)**	70.0 (45.0–87.0)	83.0 (71.0–93.0)	34.0 (23.0–48.0)	<0.0001	95.0 (93.0–99.0)	75.5 (67.0–83.0)	48.0 (42.0–55.0)	23.0 (21.0–25.0)	<0.0001
**CRP (mg/L) †**	1.8 (1.1–3.1)	1.8 (1.1–2.9)	2.4 (1.4–3.7)	0.002	2.1 (1.2–3.6)	1.7 (1.0–2.9)	2.4 (1.4–3.7)	3.0 (3.0–3.0)	0.005
**NT-proBNP (pg/mL)**	277.0 (95.0–996.3)	216.5 (91.8–834.3)	396.0 (102.3–1120.0)	0.01	240.5 (91.3–767.8)	202.5 (91.5–951.3)	349.0 (96.0–1159.0)	425.0 (145.0–1105.0)	0.08
**Coagulation and Fibrinolysis Parameters**									
**Fibrinogen (g/ L) †**	3.2 (2.6–3.9)	3.1 (2.6–3.7)	3.3 (2.6–4.1)	0.02	3.2 (2.7–3.9)	3.0 (2.5–3.7)	3.4 (2.7–4.1)	3.2 (2.6–3.9)	0.05
**PAI-1:Ag (ng/mL)**	21.4 (13.7–29.8)	20.7 (12.9–29.6)	23.5 (15.7–29.9)	0.06	19.2 (11.0–29.0)	21.6 (14.1–30.2)	18.8 (13.4–27.7)	25.2 (20.1–30.3)	0.0004
**ETP (nM ×** **min** **)**	1557.0 (1461.0–1682.3)	1541.0 (1453.0–1655.5)	1613.1 (1487.3–1782.0)	<0.0001	1556.0 (1485.5–1656.5)	1527.5 (1449.5–1655.5)	1539.0 (1448.5–1675.0)	1729.9 (1513.0–2084.0)	<0.0001
**K_s_ (×10^−9^ cm^2^)**	6.7 (6.0–7.2)	6.8 (6.1–7.4)	6.6 (5.9–7.1)	0.009	7.0 ± 0.9	6.7 ± 0.8	6.6 ± 0.9	6.5 ± 0.9	0.001
**CLT (min)**	98.0 (82.0–108.3)	95.0 (80.0–108.0)	99.0 (86.3–110.8)	0.01	90.0 (79.0–101.8)	98.5 (80.0–110.0)	98.0 (85.0–107.0)	103.0 (90.0–118.0)	<0.0001

Data are presented as mean ± standard deviation or median (interquartile range). Ag, antigen; CLT, clot lysis time; CRP, C-reactive protein; eGFR, estimated glomerular filtration rate; ETP, endogenous thrombin potential; INR, international normalized ratio; K_s_, clot permeability; NT-proBNP, N-terminal pro-B-type natriuretic peptide; PAI-1, plasminogen activator inhibitor-1. * Including 1 patient with international normalized ratio of 1.36 and liver dysfunction. † Fibrinogen and CRP concentrations were measured in 500 and 328 patients, respectively.

**Table 3:** Estimated glomerular filtration rate categories as predictors of high endogenous thrombin potential (the top quartile, ≥1682.3 nM × min, n = 125).

Adjustment	Logistic Regression Models		
	Variable	OR (95% CI)	*p* Value
No adjustment (univariate model)	eGFR categories		<0.0001
	eGFR ≥ 90 mL/min/1.73 m^2^	Reference	
	eGFR 60–89 mL/min/1.73 m^2^	1.51 (0.80–2.88)	0.21
	eGFR 30–59 mL/min/1.73 m^2^	1.59 (0.76–3.35)	0.22
	eGFR 15–29 mL/min/1.73 m^2^	8.00 (4.01–15.95)	<0.0001
Fibrinogen	eGFR categories		<0.0001
	eGFR ≥ 90 mL/min/1.73 m^2^	Reference	
	eGFR 60–89 mL/min/1.73 m^2^	1.60 (0.84–3.06)	0.15
	eGFR 30–59 mL/min/1.73 m^2^	1.47 (0.69–3.12)	0.32
	eGFR 15–29 mL/min/1.73 m^2^	8.14 (4.04–16.39)	<0.0001
CHA_2_DS_2_-VASc score	eGFR categories		<0.0001
	eGFR ≥ 90 mL/min/1.73 m^2^	Reference	
	eGFR 60–89 mL/min/1.73 m^2^	1.67 (0.87–3.20)	0.13
	eGFR 30–59 mL/min/1.73 m^2^	1.88 (0.87–4.06)	0.11
	eGFR 15–29 mL/min/1.73 m^2^	9.06 (4.44–18.46)	<0.0001
CHA_2_DS_2_-VASc score risk factors *	eGFR categories		<0.0001
	eGFR ≥ 90 mL/min/1.73 m^2^	Reference	
	eGFR 60–89 mL/min/1.73 m^2^	1.56 (0.78–3.14)	0.21
	eGFR 30–59 mL/min/1.73 m^2^	1.87 (0.81–4.30)	0.14
	eGFR 15–29 mL/min/1.73 m^2^	10.04 (4.54–22.20)	<0.0001

CI, confidence interval; eGFR, estimated glomerular filtration rate; OR, odds ratio. *Age as categorical variable: <65 vs. 65–74 vs. ≥75 years.

**Table 4:** Estimated glomerular filtration rate categories as predictors of low clot permeability (the lowest quartile, ≤6.0 × 10^−9^ cm^2^, n = 130).

Adjustment	Logistic Regression Models		
	Variable	OR (95% CI)	*p* Value
No adjustment (univariate model)	eGFR categories		0.27
	eGFR ≥ 90 mL/min/1.73 m^2^	Reference	
	eGFR 60–89 mL/min/1.73 m^2^	1.43 (0.81–2.53)	0.21
	eGFR 30–59 mL/min/1.73 m^2^	1.79 (0.93–3.42)	0.08
	eGFR 15–29 mL/min/1.73 m^2^	1.77 (0.91–3.42)	0.09
Fibrinogen	eGFR categories		0.38
	eGFR ≥ 90 mL/min/1.73 m^2^	Reference	
	eGFR 60–89 mL/min/1.73 m^2^	1.67 (0.91–3.07)	0.10
	eGFR 30–59 mL/min/1.73 m^2^	1.53 (0.76–3.09)	0.23
	eGFR 15–29 mL/min/1.73 m^2^	1.70 (0.83–3.47)	0.15
CHA_2_DS_2_-VASc score	eGFR categories		0.59
	eGFR ≥ 90 mL/min/1.73 m^2^	Reference	
	eGFR 60–89 mL/min/1.73 m^2^	1.28 (0.72–2.29)	0.40
	eGFR 30–59 mL/min/1.73 m^2^	1.47 (0.75–2.91)	0.26
	eGFR 15–29 mL/min/1.73 m^2^	1.56 (0.79–3.06)	0.20
CHA_2_DS_2_-VASc score risk factors *	eGFR categories		0.24
	eGFR ≥ 90 mL/min/1.73 m^2^	Reference	
	eGFR 60–89 mL/min/1.73 m^2^	1.56 (0.84–2.90)	0.16
	eGFR 30–59 mL/min/1.73 m^2^	1.98 (0.94–4.17)	0.07
	eGFR 15–29 mL/min/1.73 m^2^	2.02 (0.96–4.28)	0.07

CI, confidence interval; eGFR, estimated glomerular filtration rate; OR, odds ratio. * Age as categorical variable: <65 vs. 65–74 vs. ≥75 years.

**Table 5:** Estimated glomerular filtration rate categories as predictors of prolonged clot lysis time (the top quartile, ≥108.3 min, n = 125).

Adjustment	Logistic Regression Models		
	Variable	OR (95% CI)	*p* Value
No adjustment (univariate model)	eGFR categories		0.001
	eGFR ≥ 90 mL/min/1.73 m^2^	Reference	
	eGFR 60–89 mL/min/1.73 m^2^	2.42 (1.30–4.50)	0.005
	eGFR 30–59 mL/min/1.73 m^2^	1.49 (0.70–3.15)	0.30
	eGFR 15–29 mL/min/1.73 m^2^	3.61 (1.80–7.25)	0.0003
Fibrinogen	eGFR categories		0.001
	eGFR ≥ 90 mL/min/1.73 m^2^	Reference	
	eGFR 60–89 mL/min/1.73 m^2^	2.39 (1.28–4.46)	0.006
	eGFR 30–59 mL/min/1.73 m^2^	1.49 (0.70–3.16)	0.30
	eGFR 15–29 mL/min/1.73 m^2^	3.61 (1.80–7.25)	0.0003
CHA_2_DS_2_-VASc score	eGFR categories		0.002
	eGFR ≥ 90 mL/min/1.73 m^2^	Reference	
	eGFR 60–89 mL/min/1.73 m^2^	2.40 (1.28–4.51)	0.007
	eGFR 30–59 mL/min/1.73 m^2^	1.47 (0.68–3.19)	0.33
	eGFR 15–29 mL/min/1.73 m^2^	3.58 (1.76–7.28)	0.0004
CHA_2_DS_2_-VASc score risk factors *	eGFR categories		0.01
	eGFR ≥ 90 mL/min/1.73 m^2^	Reference	
	eGFR 60–89 mL/min/1.73 m^2^	2.39 (1.22–4.68)	0.01
	eGFR 30–59 mL/min/1.73 m^2^	1.26 (0.54–2.90)	0.59
	eGFR 15–29 mL/min/1.73 m^2^	2.61 (1.20–5.70)	0.02

CI, confidence interval; eGFR, estimated glomerular filtration rate; OR, odds ratio. * Age as categorical variable: <65 vs. 65–74 vs. ≥75 years.
